# The potential impact of nutritional intake on symptoms severity in patients with comorbid migraine and irritable bowel syndrome

**DOI:** 10.1186/s12883-022-02723-0

**Published:** 2022-05-30

**Authors:** Rehab Magdy, Ragaey A Eid, Mahmoud Hassan, Mohamed Abdelghaffar, Asmaa F El Sayed, Zeinab Mohammed, Mona Hussein

**Affiliations:** 1 Department of Neurology, Faculty of Medicine, Cairo University, Cairo, Egypt; 2 Department of Tropical medicine (Department of Gastroenterology, Hepatology and Endemic Medicine), Faculty of Medicine, Beni-Suef University, Beni-Suef, Egypt; 3 Department of Internal medicine, Faculty of Medicine, Beni-Suef University, Beni-Suef, Egypt; 4 Department of Neurology, Faculty of Medicine, Fayoum University, Fayoum, Egypt; 5 Department of Public Health and Community Medicine, Faculty of Medicine, Cairo University, Cairo, Egypt; 6 Department of Public Health and Community Medicine, Faculty of Medicine, Beni-Suef University, Beni-Suef, Egypt; 7 Department of Neurology, Faculty of Medicine, Beni-Suef University, Beni-Suef, Egypt

**Keywords:** Migraine, Irritable bowel syndrome, Macronutrients, micronutrients, Food frequency questionnaire

## Abstract

**Background:**

Specific dietary recommendations for migraine patients with comorbid irritable bowel syndrome (IBS) are lacking. This work aimed to study the severity scores of such two common pain-related disorders in relation to various macronutrients and micronutrients intake.

**Methods:**

A cross-sectional study was conducted on patients with concomitant migraine and IBS. The frequency and intensity of migraine attacks and the severity of IBS were evaluated. Data on dietary intake were collected using food frequency questionnaires and 24-hour dietary recall.

**Results:**

One-hundred patients with a median age of 36 years participated. The severity scores for migraine and IBS were positively correlated with fat and copper and negatively correlated with fiber and zinc intake. Copper intake was an independent predictor of the severity of both migraine and IBS (P 0.033, < 0.001). Patients with episodic migraine (*n =* 69) had a significantly higher frequency of cooked, fresh vegetables, and wheat bran bread intake (P 0.009, 0.004, 0.021) and lower frequency of hydrogenated oils intake (P 0.046), in comparison to patients with chronic migraine (*n =* 31). Patients with moderate intensity of migraine (*n =* 37) had a significantly higher frequency of herbal drinks intake (P 0.014) than patients with a severe intensity of migraine (*n =* 63). Patients with mild (*n =* 13) and moderate IBS (*n =* 41) had a significantly higher frequency of wheat bran bread and sen bread intake (P 0.003, 0.022) than patients with severe IBS (*n =* 46).

**Conclusion:**

Patients with comorbid migraine and IBS are advised to adhere to a diet low in fat and copper and rich in fiber and zinc.

**Supplementary Information:**

The online version contains supplementary material available at 10.1186/s12883-022-02723-0.

## Introduction

Several epidemiological studies have confirmed a common association between migraine and irritable bowel syndrome (IBS) [1, 2]. Migraine and IBS share specific characteristics. Both disorders are chronic pain-related, widespread among females, often accompanied by depression and anxiety, and significantly affect the patient’s quality of life [3].

The link between migraine and IBS is a good model of the brain-gut axis. The enteric nervous system is a credible common pathogenic link between migraine and IBS, as a source of various neurotransmitters, particularly serotonin [4, 5]. In addition, nutritional factors may influence the mechanisms of both disorders through their effect on gut microbiota and immune system [5].

A growing body of evidence supports that certain dietary compounds act as migraine triggers [6]. Nutritional factors also play a key role in aggravating IBS symptoms [7]. Hence, abundant scientific research has been launched on dietary management as a novel therapeutic strategy in IBS [8] as well as in migraine cases [6].

However, no study has previously examined nutrients that may affect either of these diseases at the expense of the other. Even if these factors had been studied separately for each disease, they might have a different effect when the two diseases combined in one individual.

This work aimed to study the effect of macronutrients and micronutrients in relation to severity scores of both migraine and IBS. If the food triggers were precisely identified, avoiding them will, in turn, improve the quality of life in patients with comorbid migraine and IBS.

## Methods

### Study design and participants

This cross-sectional study was carried out on 100 adults with comorbid migraine and IBS from 1 January to 1 June 2021. Patients’ recruitment was temporarily held during the holy month of Ramadan (13 April-12 May) and completed after that to avoid the exacerbating effect of fasting on migraine [9].

Diagnosis of migraine was established according to The International Classification of Headache Disorders, 3rd edition [10], while IBS diagnosis was made according to The Rome IV criteria [11]. Patients aged ≥18 years, of both sexes, who could appropriately respond to the questionnaires were eligible. The patients were consecutively recruited from three centres nationwide, the University Hospitals in Beni-Suef, Fayoum, and Cairo.

Exclusion criteria included other comorbidities that may affect the nutritional status significantly (ex, malignancy, chronic kidney disease, chronic infections, malabsorption syndromes, etc.), an endocrine disorder causing alterations in energy metabolism (diabetes, thyroid dysfunction), pregnant or lactating women, patients on dietary supplements, and the use of prophylactic migraine treatment.

### Data collection tools

All patients who met the eligibility criteria were submitted to the following:

### Clinical assessment

All patients underwent an integrated evaluation by the research team, first by a gastroenterologist who conducted thorough medical history and examination to confirm the diagnosis of IBS after exclusion of any red signs according to The Rome IV criteria [11]. According to the criteria mentioned above, IBS was classified into four categories: IBS with predominant constipation, IBS with predominant diarrhea, IBS with mixed bowel habits, and unclassified IBS. The severity of IBS symptoms was assessed using The irritable bowel severity scoring system (IBS-SSS) [12], with a maximum achievable score of 500. The grading severity of IBS was indicated according to the total score as follows: mild (75 to 175), moderate (> 175 to 300), and severe cases by scores > 300 [12].

Then the neurologist proceeded in a comprehensive headache assessment, including frequency, duration, and intensity of untreated headache attacks according to the visual analogue scale (VAS). The grading intensity of migraine was determined according to the total score of VAS as follows: mild (1 to 3), moderate (4 to 6), and severe cases by scores > 7 [13].

### Nutritional assessment

The patients were contacted after a diagnosis of comorbid migraine and IBS were confirmed. A face-to-face interview was scheduled with an expert nutritionist within a week of patients’ recruitment. The nutritional evaluation included:

#### *Food frequency questionnaire (FFQ)* [14]

For the FFQ, each participant was asked to choose an answer expressing the rate of his usual intake of 32 food items grouped into five large groups: fruits and vegetables, bread and cereals, proteins, fats, and miscellaneous foods (Additional file 1). Response categories were as follows: 0-2 times / week, 3-4 times / week, or ≥ 5 times / week.

#### *The 24-hour dietary recall* [15]

During this interview, patients were asked to record the amounts they ingested the previous day. Data on the food preparation method, ingredients in mixed dishes, the brand name of different commercial products, and quantities of each food and beverage consumed were collected comprehensively in a pre-designed form.

Each food consumed was estimated using household measures of a standard size (e.g., cups, spoons, and mugs), then converted to grams. Some visual aids were also used to help patients accurately report serving size. This interview required about 20 to 30 minutes to be completed.

After that, the dietitian phoned patients to evaluate their consumed food for the other 2 days, one of them on the weekend.

After collecting data for these 3 days of the 24-hour dietary recall, food consumed was converted into nutrients intake in international units using the Nutrition Data System for Research (NDS) software [16] at the National Institute of Nutrition in Cairo.

### Sampling

The sample size was calculated using Epi info, version 3.5.1, 2008. Based on a confidence level of 95 and 6% prevalence rate of migraine in IBS [2], a total sample size of at least 86 patients was required to achieve a statistical power of 80%.

### Ethical statement

Written informed written consent was signed from all included patients. The study was performed following the Declaration of Helsinki. Ethical approval was obtained from the Research ethical committee of Beni-Suef University. The ethical committee approval number was FMBSUREC/06042021/Hussein-3.

### Statistical analysis

IBM SPSS (Statistical Package of Social Science) Version 26 was used to analyze the data. Kolmogorov–Smirnov test was used to test the normality of data. Categorical variables in patients’ demographics and clinical characteristics were expressed as numbers and percentages. Non-normally distributed quantitative variables in the demographics, clinical characteristics, and nutritional assessment were expressed as the median and interquartile range (IQR). Correlations between severity scores of both migraine and IBS, and 24-hour dietary recall items were done using Spearman correlation test. A chi-squared test was used to compare patients in the food frequency questionnaire items. Multivariate linear regression analysis was used to identify dietary predictors of frequency and intensity of migraine attacks and severity of IBS. A *P-*value less than 0.05 was considered statistically significant. All tests were two-tailed.

## Results

### Demographics and clinical characteristics of the included patients

This cross-sectional study was carried out on 100 adults with comorbid migraine and IBS. The median age was 36 years with an interquartile range (29.5- 44). Demographics and clinical characteristics of migraine and IBS were demonstrated in Table 1.

**Table 1:** Demographics and clinical characteristics of the included patients

	Patients (*n =* 100)
Age [Median (IQR)]	36 (29.5- 44)
Sex	
Males [n (%)]	27 (27%)
Females [n (%)]	73 (73%)
BMI [Median (IQR)]	26.12 (23.42- 29.4)
Age at onset of migraine [Median (IQR)]	25 (20- 32)
Type of Migraine	
Episodic [n (%)]	69 (69%)
Chronic [n (%)]	31 (31%)
Aura	
With aura [n (%)]	32 (32%)
Without aura [n (%)]	68 (68%)
Frequency of migraine attacks/ month [Median (IQR)]	8 (4 - 20)
VAS [Median (IQR)]	7 (5-8)
Duration of untreated attack in hours [Median (IQR)]	6 (4- 24)
Abortive treatment	
Paracetamol [n (%)]	32 (32%)
NSAIDs [n (%)]	31 (31%)
Triptans [n (%)]	19 (19%)
Combined [n (%)]	18 (18%)
Response to Abortive treatment	
Good [n (%)]	35 (35%)
Moderate [n (%)]	40 (40%)
Poor [n (%)]	25 (25%)
Age at onset of IBS [Median (IQR)]	24 (20- 30)
Type of IBS	
Constipation -predominant [n (%)]	36 (36%)
Diarrhea -predominant [n (%)]	10 (10%)
Unclassified -IBS [n (%)]	25 (25%)
Mixed -diarrhea-and-constipation [n (%)]	29 (29%)
IBS severity scoring system [Median (IQR)]	300 (222.5- 400)

*BMI* Body mass index, *IBS* Irritable bowel syndrome, *IQR* Interquartile range, *VAS* Visual analogue scale

### Effect of macronutrients and micronutrients intake on frequency, the intensity of migraine attacks, IBS type and IBS severity

The median values of the macronutrients and micronutrients analyzed from the 24-hour dietary recall questionnaire were demonstrated in Table 2.

**Table 2:** Results of 24 hours dietary recall

	Patients (*n =* 100) [Median (IQR)]
**Macronutrients**	
Energy (K Calories)	1665.33 (1232.91- 1982.58)
Proteins (grams)	55.84 (39.95- 72.35)
Fats (grams)	45.67 (26.19- 76.49)
Fibers (grams)	6.4 (3.85- 9.1)
Carbohydrates (grams)	239.76 (184.93- 295.16)
**Micronutrients**	
Sodium (mg)	1918.83 (1359.2- 3600.35)
Potassium (mg)	2015.52 (1508.51- 2512.83)
Calcium (mg)	444.8 (299.3- 605.1)
Phosphorus (mg)	839.36 (604.57- 1117.11)
Magnesium (mg)	95.70 (66.90- 126.95)
Iron (mg)	12.07 (8.79- 15.67)
Zinc (mg)	7.65 (5.2- 10.66)
Copper (mg)	1.07 (0.58- 1.52)
Vitamin A (μg)	164.02 (88.76- 297.43)
Vitamin C (mg)	46.76 (9.3- 89.17)
Vitamin B1 (mg)	0.83 (0.54 - 1.08)
Vitamin B2 (mg)	0.59 (0.37-0.78)

Regarding the macronutrients, there were statistically significant negative correlations between dietary fiber and each frequency of migraine/month and IBS-SSS (*P-*value 0.003, < 0.001, respectively). Also, dietary fats were positively correlated with each frequency of migraine/month, VAS, and IBS-SSS (< 0.001, < 0.001, 0.001, respectively) (Table 3).

**Table 3:** Correlations between macronutrients and micronutrients intake and frequency, intensity of migraine attacks, and IBS severity scoring system

	Frequency of migraine attacks / month		VAS		IBS severity scoring system	
	(r) coef.	*P-* value	(r) coef.	*P-* value	(r) coef.	*P-* value
K Calories	−0.169	0.093	−0.057	0.575	0.028	0.780
Proteins	−0.165	0.101	−0.039	0.703	0.002	0.985
Fats	0.393	< 0.001*	0.387	< 0.001*	0.327	0.001*
Fibers	−0.294	0.003*	−0.139	0.170	−0.380	< 0.001*
Carbohydrates	−0.116	0.249	−0.050	0.621	0.044	0.661
Sodium	0.425	< 0.001*	0.525	< 0.001*	−0.073	0.472
Potassium	−0.009	0.925	0.050	0.619	0.036	0.722
Calcium	0.062	0.540	0.124	0.218	0.000	0.999
Phosphorus	−0.142	0.160	0.062	0.539	−0.052	0.608
Magnesium	−0.075	0.461	−0.002	0.981	0.173	0.085
Iron	−0.133	0.187	0.000	0.999	−0.028	0.781
Zinc	−0.677	< 0.001*	−0.442	< 0.001*	− 0.220	0.028*
Copper	0.283	0.004*	0.088	0.385	0.706	< 0.001*
Vitamine A	0.021	0.837	0.072	0.072	−0.075	0.455
Vitamine C	−0.204	0.046*	−0.084	0.416	−0.026	0.799
Vitamine B1	−0.083	0.411	0.024	0.809	−0.020	0.841
Vitamine B2	−0.055	0.584	0.040	0.690	0.030	0.770

*IBS* Irritable bowel syndrome, *VAS* Visual analogue scale

*P-value ≤0.05 is considered significant

For the micronutrients, dietary zinc was negatively correlated with each frequency of migraine/month, VAS, and IBS-SSS (< 0.001, < 0.001, 0.028, respectively). Also, dietary copper was positively correlated with each frequency of migraine/month and IBS-SSS (0.004, < 0.001, respectively) (Table 3).

On the other hand, dietary sodium was positively correlated with the frequency of migraine/month and VAS, while vitamin C was negatively correlated with the frequency of migraine/month (Table 3). Nevertheless, none of these nutrients was significantly correlated with IBS-SSS.

Patients with diarrhoea-predominant IBS had a significantly higher intake of fibers and Zinc than those with constipation-predominant IBS (*P-*value = 0.047, 0.006, respectively) (Table 4).

**Table 4:** Comparison between constipation-predominant and diarrhoea-predominant IBS regarding macronutrients and micronutrients

	Patients with constipation-predominant IBS (*n =* 36) [Median (IQR)]	Patients with diarrhea-predominant IBS (*n =* 10) [Median (IQR)]	*P-*value
**Macronutrients**			
Energy (K Calories)	1474.93 (845.9-2464.97)	1835.95 (11.34-2148.41)	0.452
Proteins (grams)	57.26 (28.32-81.75)	64.23 (31.38-81.28)	0.606
Fats (grams)	76.18 (68.22-87.61)	46.6 (21.31-77.24)	0.104
Fibers (grams)	4.8 (3.5- 6.1000)	7.25 (5.35- 9.1)	0.047*
Carbohydrates (grams)	237.51 (143.65- 362.88)	208.85 (160.78-308.88)	0.663
**Micronutrients**			
Sodium (mg)	1706.92 (1359.81-4123.8)	1504.71 (1237.5-2889.12)	0.405
Potassium (mg)	2294.65 (1232.7-3080.65)	2057.99 (1454.3-2167.39)	0.968
Calcium (mg)	435.9 (314.2-708.2)	439.95 (320.83-675.65)	0.843
Phosphorus (mg)	815.55 (493.2-1245.73)	865.55 (598.48-1203.31)	0.552
Magnesium (mg)	104.7 (50.8-132.5)	98.9 (57.58-116.08)	0.782
Iron (mg)	11.55 (8.58-18.35)	11.95 (8.32-16.48)	0.606
Zinc (mg)	5.05 (4.14-9.06)	10.84 (9.75-17.07)	0.006*
Copper (mg)	1.43 (1.2-1.53)	0.78 (0.39-1.44)	0.165
Vitamin A (μg)	143.5 (81.9-319.65)	256.1 (72.13-1156.12)	0.606
Vitamin C (mg)	22.43 (8.7-46.76)	73.37 (3.78-80.04)	0.513
Vitamin B1 (mg)	0.91 (0.49-1.08)	0.88 (0.35-1.21)	0.606
Vitamin B2 (mg)	0.54 (0.38-1.06)	0.56 (0.36- 0.96)	0.634

*IBS* Irritable bowel syndrome

*P-value ≤0.05 is considered significant

### Effect of frequency of different food items intake on frequency and intensity of migraine attacks, and IBS severity

Among the fruits and vegetable category, only cooked and fresh vegetable intake was significantly higher in patients with episodic migraine (*n =* 69) in comparison to patients with chronic migraine (*n =* 31) (*P-*value = 0.009, 0.004), (Table 5). On the other hand, none of these category items was significantly different between mild (*n =* 10), moderate (*n =* 44), and severe IBS (*n =* 46).

**Table 5:** Results of food frequency questionnaire in relation to migraine type and severity

		Migraine type			Migraine intensity		
		Episodic migraine 69 (69%)	Chronic migraine 31 (31%)	*P-*value	Moderate 37 (37%)	Severe 63 (63%)	*P-*value
Cooked vegetables	0-2 times/week	26 (37.7%)	17 (54.8%)	0.009*	14 (37.8%)	29 (46.0%)	0.724
	3 - 4 times/week	26 (37.7%)	14 (45.2%)		16 (43.2%)	24 (38.1%)	
	≥ 5 times/week	17 (24.6%)	0 (0%)		7 (18.9%)	10,915.9%)	
Fresh vegetables	0-2 times/week	35 (50.7%)	18 (58.1%)	0.004*	22 (59.5%)	31 (49.2%)	0.229
	3 - 4 times/week	16 (23.2%)	13 (41.9%)		7 (18.9%)	22 (34.9%)	
	≥ 5 times/week	18 (26.1%)	0 (0%)		8 (21.6%)	10 (15.9%)	
Wheat bran bread	0-2 times/week	15 (21.7%)	13 (41.9%)	0.021*	11 (29.7%)	17 (27%)	0.555
	3 - 4 times/week	9 (13.0%)	7 (22.6%)		4 (10.8%)	12 (19%)	
	≥ 5 times/week	45 (65.2%)	11 (35.5%)		22 (59.5%)	34 (54.0%)	
Rice and pasta	0-2 times/week	25 (36.2%)	12 (38.7%)	0.04*	11 (29.7%)	26 (41.3%)	0.481
	3 - 4 times/week	22 (31.9%)	16 (51.6%)		15 (40.5%)	23 (36.5%)	
	≥ 5 times/week	22 (31.9%)	3 (9.7%)		11 (29.7%)	14 (22.2%)	
Red meat	0-2 times/week	33 (47.8%)	21 (67.7%)	0.044*	17 (45.9%)	37 (58.7%)	0.220
	3 - 4 times/week	26 (37.7%)	10 (32.3%)		14 (37.8%)	22 (34.9%)	
	≥ 5 times/week	10 (14.5%)	0 (0%)		6 (16.2%)	4 (6.3%)	
Poultry	0-2 times/week	35 (50.7%)	22 (71%)	0.037*	17 (45.9%)	40 (63.5%)	0.091
	3 - 4 times/week	23 (33.3%)	9 (29%)		13 (35.1%)	19 (30.2%)	
	≥ 5 times/week	11 (15.9%)	0 (0%)		7 (18.9%)	4 (6.3%)	
Hydrogenated oils	0-2 times/week	52 (75.4%)	24 (77.4%)	0.046*	29 (78.4%)	47 (74.6%)	0.877
	3 - 4 times/week	12 (17.4%)	1 (3.2%)		4 (10.8%)	9 (14.3%)	
	≥ 5 times/week	5 (7.2%)	6 (19.4%)		4 (10.8%)	7 (11.1%)	
Herbal drinks	0-2 times/week	44 (63.8%)	26 (83.9%)	0.092	22 (59.5%)	48 (76.2%)	0.014*
	3 - 4 times/week	14 (20.3%)	4 (12.9%)		6 (16.2%)	12 (19.0%)	
	≥ 5 times/week	11 (15.9%)	1 (3.2%)		9 (24.3%)	3 (4.8%)	

*P-value ≤0.05 is considered significant

Regarding bread and cereals category, frequency of wheat bran bread intake was significantly higher in patients with episodic than those with chronic migraine (*P-*value = 0.021) (Table 5) as well as in patients with mild and moderate IBS than those with severe IBS (*P-*value =0.003), (Table 6). From the same food category, it was found that patients with episodic migraine had a significantly higher frequency of rice and pasta intake (*P-*value = 0.04) than patients with chronic migraine (Table 5). In addition, patients with mild IBS had a significantly higher frequency of sen bread intake than patients with moderate and severe IBS (*P-*value =0.022) (Table 6).

**Table 6:** Results of food frequency questionnaire in relation to IBS severity

		IBS severity scoring system			
		Mild 10 (10%)	Moderate 44 (44%)	Severe 46 (46%)	*P-*value
Wheat bran bread	0-2 times/week	1 (10%)	11 (25%)	16 (34.8%)	0.003*
	3 - 4 times/week	1 (10%)	2 (4.5%)	13 (28.3%)	
	≥ 5 times/week	8 (80%)	31 (70.5%)	17 (37%)	
Sen bread	0-2 times/week	9 (90%)	44 (100%)	44 (95.7%)	0.022*
	3 - 4 times/week	0 (0%)	0 (0%)	2 (4.3%)	
	≥ 5 times/week	1 (10%)	0 (0%)	0 (0%)	

*IBS* Irritable bowel syndrome,

*P-value ≤0.05 is considered significant

In the case of the proteins category, only red meat and poultry intake were significantly higher in patients with episodic than those with chronic migraine (*P-*value = 0.044 and 0.037), respectively (Table 5). Yet, none of these food items was significantly different between different groups of IBS severity.

For the fats group, only hydrogenated oils intake was significantly lower consumed in patients with episodic than chronic migraine (*P-*value = 0.046) (Table 5), with no significant difference between the different groups of IBS severity.

The last food category in FFQ, “Miscellaneous foods,” showed that patients with moderate intensity of migraine (*n =* 37) had a significantly higher frequency of herbal drinks intake in comparison to patients with a severe intensity of migraine (*n =* 63) (*P-*value =0.014) (Table 5).

### Dietary predictors of frequency and intensity of migraine attacks and severity of IBS

Multivariate linear regression analysis was done to determine dietary predictors of frequency and intensity of migraine attacks and severity of IBS. The following variables were used as independent variables in the regression model: dietary fat, fiber, zinc, and copper.

Higher intake of fat and a lower intake of zinc were independent predictors of increased frequency and intensity of migraine attacks (*P-*value = 0.004, < 0.001, < 0.001, < 0.001, respectively). Lower intake of fibers was an independent predictor of increased severity of IBS (*P-*value < 0.001), and higher intake of copper was an independent predictor of increased intensity of migraine attacks and severity of IBS (*P-*value = 0.033, < 0.001, respectively) (Table 7).

**Table 7:** Multivariate linear regression analysis to detect dietary predictors of frequency and intensity of migraine attacks, and severity of IBS

Dependent variables	Independent variables	B	*P-*value	95% Confidence Interval		Adjusted R Squared
				Lower bound	Upper bound	
Frequency of migraine attacks/ month	Fat	0.085	0.004*	0.027	0.142	0.390
	Fiber	−0.184	0.501	−0.725	0.357	
	Zinc	−0.841	< 0.001*	−1.150	−0.532	
	Copper	1.523	0.296	−1.353	4.398	
VAS	Fat	0.023	< 0.001*	0.012	0.035	0.365
	Fiber	0.045	0.420	−0.066	0.156	
	Zinc	− 0.190	< 0.001*	− 0.253	− 0.126	
	Copper	−0.642	0.033*	−1.230	−0.054	
IBS severity scoring system	Fat	−0.194	0.495	−0.758	0.369	0.587
	Fiber	−10.99	< 0.001*	− 16.33	−5.637	
	Zinc	2.526	0.104	−0.525	5.577	
	Copper	137.43	< 0.001*	109.03	165.84	

*IBS* Irritable bowel syndrome, *VAS* Visual analogue scale

*P-value ≤0.05 is considered significant

## Discussion

Studying the influential role of diet in individuals with concomitant migraine and IBS opens the way for designing unique therapeutic diet regimens that may improve the quality of life for these patients, in particular, after the emergence of many dietary interventions that have proven therapeutic success in each of the two diseases separately [6]. To our knowledge, this is the first work that studied the severity scores of such common pain-related disorders in relation to various macronutrients and micronutrients.

In the current study, the severity scores for both disorders significantly increased with increased dietary intake of fats and copper and decreased intake of fibers and zinc. On the other hand, increased sodium and decreased vitamin C intake showed only an association with increased frequency or intensity of migraine with no association with the IBS-severity.

Regarding the dietary lipids, this study agreed with LA Ferrara, D Pacioni, V Di Fronzo, BF Russo, E Speranza, V Carlino, F Gargiulo and F Ferrara [17], who found that the frequency and intensity of migraine attacks were significantly reduced with a low-lipid diet as well as with C Feinle-Bisset and F Azpiroz [18], who reported the same relieving effect on IBS symptoms. Such a common effect may be explained by the potential lowering effect in serotonin- a common major player in both disorders- after a fat-rich meal [19].

Yet, many types of dietary fats differ in their chemical components and thus in their effect on health. For instance, the hydrogenated oils are rich in saturated fats and trans fats [20], consumed more in our patients with chronic migraine than episodic type. In addition, the hydrogenation process itself reduces omega-3 fat intake [21]. Hence, intake of omega-3 fatty acids has successfully reduced migraine frequency and intensity [22].

Large evidence exists that IBS-associated symptoms are highly aggravated by a deficient dietary fibre intake [23]. This study demonstrated this relation with IBS severity and with migraine frequency and intensity. K Makki, EC Deehan, J Walter and F Bäckhed [24] reported the beneficial effects of high dietary fiber by replenishing the gut microbiome with essential missing microbes.

The total dietary fiber content in whole-grain bread (either wheat bran bread or sin bread) is much higher (40-44%) than the white flour bread (2.5%), thus making it an ideal supplement for producing rich-fiber baked products [25]. The present study proved that the consumption of whole-grain bread was significantly higher in patients with episodic than chronic migraine and patients with mild IBS than in the severe form.

Vegetables as another example of a high-fibre diet. Their consumption was significantly greater in our patients with episodic than chronic migraine, regardless of whether they were fresh or cooked. As the cooking\heating process doesn’t destroy vegan fibres [26], migraine patients have more opportunities to eat whatever they like.

Although several micronutrients have been documented in the pathogenesis of both migraine [27] and IBS [28], the only trace elements identified in this study to have a common relationship between the two diseases are dietary zinc and copper. Notably, an elevated copper-zinc ratio is a well-known marker of increased inflammation and oxidative stress [29]. In line with our findings, some studies found that higher copper and lower zinc might trigger migraine headaches [30, 31] and IBS-related symptoms [28]. That is why it was noted in this study that the consumption of herbal drinks was associated with a reduction in the intensity of migraine, as the herbal drinks frequently used by Egyptians (mint, anise, parsley, roselle, and chamomile) are characterized by a high nutritional content of zinc [32, 33]. Other examples of a high-zinc, low-copper diet include beef, poultry, eggs, and cheese/cheese products [34].

This study also observed that high dietary sodium and low vitamin C aggravated the number and intensity of migraine attacks. Other studies similarly proved the preventive effects of a low sodium diet [35] and a combined antioxidant regimen including vitamin C [36]. Yet, neither of these micronutrients was associated with the severity of the IBS in this study.

Some limitations of the current study are worth mentioning. Our results primarily relied on what patients recalled about their dietary intake. Biochemical analysis of various micronutrients has not been performed to provide a more objective evaluation. Other drawbacks of the study are its cross-sectional design and the absence of a control group for comparison. Further prospective studies are encouraged to avoid recall bias. Furthermore, dietary habits vary greatly worldwide, affecting the nutritional assessment and hindering the generalizability of our results. Designing food frequency questionnaires that include Mediterranean and Western diets may help overcome such limitations.

## Conclusion

Migraine patients with comorbid IBS are counselled to adhere to a low-fat and high-fibre diet. A high-zinc and low-copper diet should also be fortified. The patients should be encouraged to increase their intake of cooked and fresh vegetables, whole-grain bread, herbal drinks and to avoid hydrogenated oils as much as possible. These specific dietary recommendations may efficiently attenuate the symptoms of both disorders.

## Supplementary Information

**Additional file 1.**

## Data Availability

The datasets used and/or analyzed during the current study are available from the corresponding author on reasonable request.

## Abbreviations

IBS: Irritable bowel syndrome
IBS-SSS: The irritable bowel severity scoring system
VAS: Visual analogue scale
FFQ: Food frequency questionnaire
NDS: Nutrition Data System for Research
IQR: Interquartile range
