# Supplementary material for: The potential impact of nutritional intake on symptoms severity in patients with comorbid migraine and irritable bowel syndrome
Source: BMC Neurol. 2022 May 30;22:199. doi: 10.1186/s12883-022-02723-0 (PMC9150376; doi:10.1186/s12883-022-02723-0)
Supplement: Supplementary file 1 — Additional file 1. [file 12883_2022_2723_MOESM1_ESM.docx]

**Food Frequency Questionnaire**

| **Food item** | 0-2 times/week | 3 - 4 times/week | ≥ 5 times/week |
| --- | --- | --- | --- |
| **Fruits and vegetables**  Cooked vegetables  Fresh vegetables  Leaves vegetables  Fresh fruits  Canned juices |  |  |  |
| **Bread and cereals**  Wheat bran bread  White bread  Other bakeries  Sin bread  Rice & Pasta |  |  |  |
| **Proteins**  Milk  Natural cheeses  Processed cheeses  Red meats  Poultry  Processed meats  Fish  Salty fish  Eggs  Legumes |  |  |  |
| **Different types of fats**  Oils  Olive oil  Butter  Hydrogenated oils |  |  |  |
| **Miscellaneous foods**  Soft drinks  Hot caffeinated drinks  Herbal dinks  Crackers  Nuts  Pumpkin seed  Pickles  Salt |  |  |  |
